# Immunoregulatory functions and therapeutic potential of natural killer cell-derived extracellular vesicles in chronic diseases

**DOI:** 10.3389/fimmu.2023.1328094

**Published:** 2024-01-03

**Authors:** Shuang He, Lanqian Su, Haiyang Hu, Haiqi Liu, Jingwen Xiong, Xiangjin Gong, Hao Chi, Qibiao Wu, Guanhu Yang

**Affiliations:** ^1^ Faculty of Chinese Medicine, and State Key Laboratory of Quality Research in Chinese Medicine, Macau University of Science and Technology, Macao, Macao SAR, China; ^2^ Clinical Medical College, Southwest Medical University, Luzhou, China; ^3^ Department of Sports Rehabilitation, Southwest Medical University, Luzhou, China; ^4^ Department of Specialty Medicine, Ohio University, Athens, OH, United States

**Keywords:** NK cell, NK cell-derived exosome, engineered EVs, chronic disease, microvesicles, therapy, surface modification

## Abstract

Extracellular vesicles (EVs) have been proven to play a significant immunoregulatory role in many chronic diseases, such as cancer and immune disorders. Among them, EVs derived from NK cells are an essential component of the immune cell functions. These EVs have been demonstrated to carry a variety of toxic proteins and nucleic acids derived from NK cells and play a therapeutic role in diseases like malignancies, liver fibrosis, and lung injury. However, natural NK-derived EVs (NKEVs) have certain limitations in disease treatment, such as low yield and poor targeting. Concurrently, NK cells exhibit characteristics of memory-like NK cells, which have stronger proliferative capacity, increased IFN-γ production, and enhanced cytotoxicity, making them more advantageous for disease treatment. Recent research has shifted its focus towards engineered extracellular vesicles and their potential to improve the efficiency, specificity, and safety of disease treatments. In this review, we will discuss the characteristics of NK-derived EVs and the latest advancements in disease therapy. Specifically, we will compare different cellular sources of NKEVs and explore the current status and prospects of memory-like NK cell-derived EVs and engineered NKEVs.

## 1 Introduction

Extracellular vesicles are vesicles enclosed by phospholipid bilayers secreted by all cell types, so they can be found in tissue culture supernatants and biological fluids, such as blood, saliva, breast milk, cerebrospinal fluid, and malignant ascites (1). Based on the different formation processes, EVs were divided into three groups: exosomes, microvesicles (microparticles) and apoptotic bodies. Among them, exosomes have received more attention. They are EVs with a size range of 50~200nm (2). They bud inward from the limiting membrane of endosomes to form multivesicular bodies (MVBs). Subsequently, MVBs fuse with the plasma membrane to release exosomes into the extracellular space. The surface of exosomes is enriched with tetraester proteins, such as CD63, CD81 and CD9. Although exosomes have no final and specific surface markers, they are a combination of expression markers and lack of specific intracellular protein expression, meeting the minimum requirements of the current exosome definition. Due to the overlap size between the three populations, surface markers, and the lack of proteins restricted to specific populations,it has been a challenge to distinguish exosomes from microvesicles (3); At present, researchers collectively refer to three different types of vesicles as EVs.

Natural killer (NK) cells are natural lymphocytes that fight infection and kill tumor cells, mainly in the peripheral blood, bone marrow, lymph nodes, and spleen (4). Based on the recognition of activating or inhibitory receptors and stress-induced ligands, NK cells not only enhance cytokine production and cell killing, but also provide immune self-tolerance and negative feedback mechanisms, and perform the three major functions of immune surveillance, immune response, and immune memory (5, 6).

Another mechanism of NK cell involves rapidly killing target cells through the slow Fas-FasL-dependent pathway, or via the utilization of intracellular lytic granules releasing proteins such as granzymes and perforin (7). The formidable antineoplastic potential of NK cells has been effectively leveraged in numerous clinical trials, employing autologous or allogeneic NK cells, as well as chimeric antigen receptor (CAR)-modified NK cells, in the concerted effort to combat hematologic malignancies (8–10). Nonetheless, the therapeutic application of NK cells in the context of solid tumors poses a more intricate challenge, primarily attributed to their constrained capacity for infiltrating neoplastic tissues (11, 12). Considering the unique biocompatibility and higher penetration ability of EVs, NK cell-derived EVs may be the key to overcoming this challenge.

In the burgeoning domain of targeted drug delivery, the realm of nanotechnology has emerged as a pivotal contributor, notably through the advancement of intelligent carriers. Among these carriers, systems predicated on EVs have garnered considerable and pervasive attention (12). EVs are crucial mediators in many physiological processes, and EVs derived from NK cells can inherit bioactive molecules and some membrane proteins from parent cells, playing a role in immune surveillance and cytotoxicity. They also serve as carrier systems that effectively target solid tumor cells, playing a significant role in the treatment of cancer, metabolic, and neurodegenerative diseases (13, 14). However, achieving efficient and precise drug delivery for specific applications of NKEVs presents significant challenges. Engineering of isolated NKEVs through genetic engineering or chemical modifications can effectively enhance their targeting ability, homing and chemotaxis, as well as their immunomodulatory and anti-tumor capabilities. Although NK cell-derived extracellular vesicles have not yet entered clinical trials, they have become an important research focus (14).

## 2 Basic biology of NK cell-derived extracellular vesicles

### 2.1 Components and mechanisms of action

#### 2.1.1 Cytotoxic proteins

**Figure 1:**
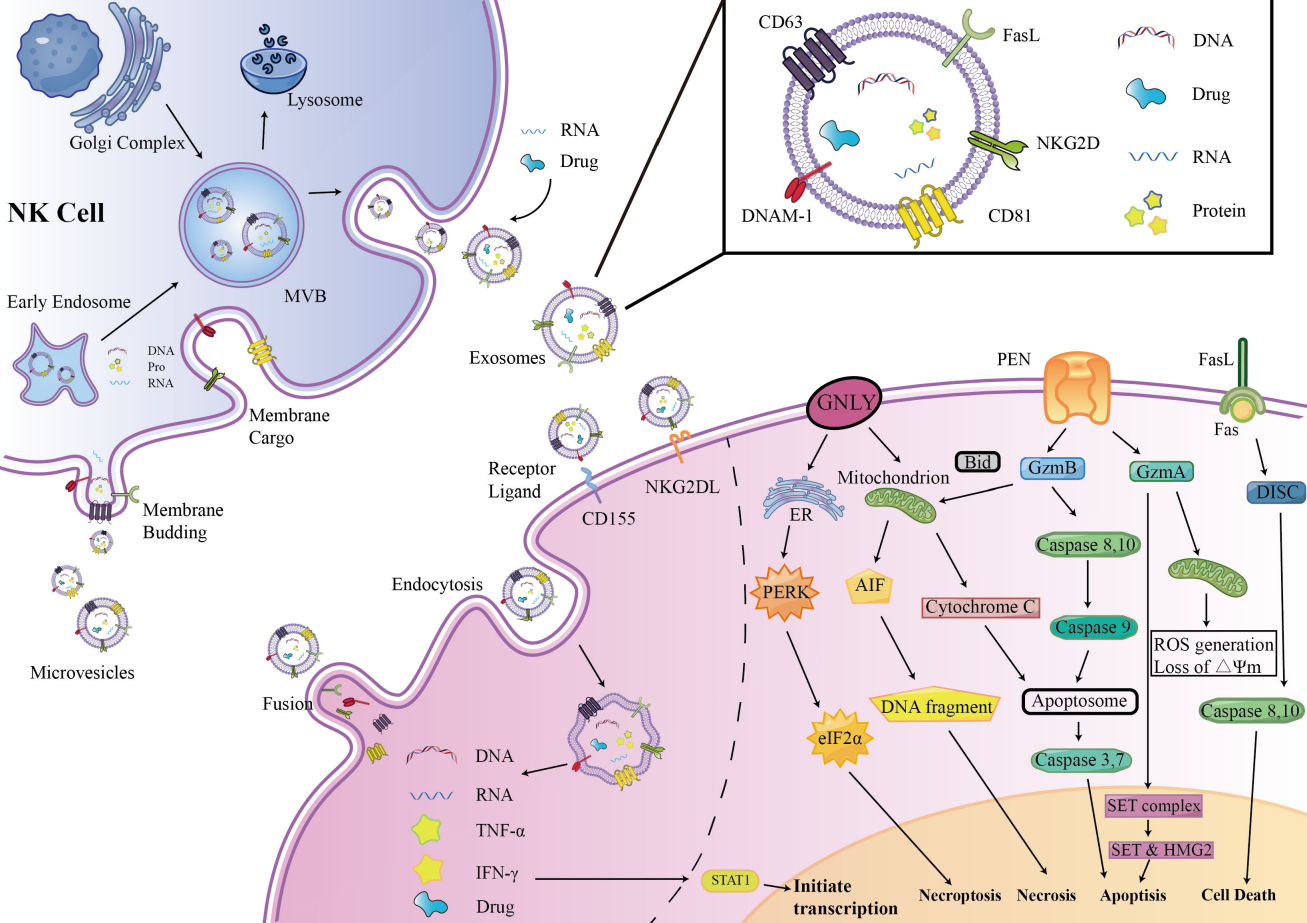
NKEVs biogenesis, secretion, content, uptake mechanism and Cytotoxic effects. EVs were divided into three groups: exosomes, microvesicles (microparticles) and apoptotic bodies. NK cell-derived EVs express activation receptors eg. NKG2D and DNAM1, and express molecules involved in cytotoxicity, e.g., perforin-granzyme mediated fusion, receptor–ligand mediated reaction, granulysin mediated electrostatic interaction.

Enriched cytotoxic proteins from NK cell sources are a typical characteristic of NKEVs and a key mechanism by which NKEVs exert cytotoxic effects. These proteins mainly include perforin (PFN), granzyme A (GzmA), granzyme B (GzmB), and granulysin (GNLY) (**Figure 1**).

Perforin is a pore-forming protein that can create pores on the endosome membrane, releasing granzyme B into the target cell, subsequently inducing target cell death through apoptosis (15). The levels of perforin in NKEVs are exceptionally high, several times to several tens of times higher than other cytotoxic proteins (16).

GzmA is a serine protease that induces cysteine-dependent apoptosis (17). Upon entering the nucleus of target cells, GzmA cleaves the SET complex, shifting it from DNA repair to DNA damage (18). As integral constituents of the SET complex, SET and HMG2 proteins assume the role of substrates for GzmA in the context of programmed cell death. Evidently, the degradation of SET and HMG2B becomes apparent in CHLA255 and SupB15 cells upon treatment with NKEVs (16). Additionally, GzmA orchestrates the activation of a pathway intricately linked to mitochondrial stress. Upon internalization into target cells, GzmA instigates oxidative stress reactions, instigating mitochondrial depolarization and a surge in reactive oxygen species (ROS). This cascade, in turn, facilitates the aggregation of the SET complex within the cellular nucleus (19, 20). Consequently, GzmA elicits a distinctive and parallel cell death pathway, operating autonomously of caspases.

GzmB is possibly the most active member of the granzyme family. After entering the cytoplasm of target cells, GzmB activates initiator caspases, caspase-8 and caspase-10, following two pathways to promote cell apoptosis (17). One is directly processing of caspase-3, 7 by GzmB, thus promoting cell apoptosis. The other pathway is related to mitochondrial-associated caspase cascades. GzmB promotes the release of cytochrome C (cyt c) from the mitochondrial membrane interstitial space into the cytoplasm to bind to caspase- 9 and form apoptotic vesicles by truncating BID, which indirectly promotes the activation of caspase-3, 7 (16, 21, 22). Wu et al. found that NK-EVs can induce the release of cytochrome c from neuroblastoma cells, confirming the mechanism by which GzmB in NKEVs exerts its cytotoxicity. GzmB and GNLY may also induce cell death in target cells through endoplasmic reticulum stress via NK-EVs (16).

FasL is a type II transmembrane protein of the tumor necrosis factor superfamily, which interacts with receptors on the target cell membrane, such as Fas or CD95, leading to receptor trimerization. Subsequently, a death-inducing signaling complex (DISC) is formed, recruiting and activating caspase-8,10 proenzymes, promoting cell apoptosis through two pathways (23, 24). In one pathway, a large amount of caspase-8 proenzymes is recruited, activating the caspase-3 and caspase-7 pathways. In the other pathway, a small amount of caspase-8 cleaves BID, activating the mitochondria-related apoptosis pathway (25, 26).

The involvement of FasL in the cytotoxicity exerted by NKEVs has engendered substantial discourse within the scientific community. Divergent perspectives posit potential mechanisms, with one school of thought implicating classical receptor-ligand interactions facilitated by FasL-expressing NK92-cell-derived EVs. This notion gains empirical support as these vesicles demonstrate time- and dose-dependent cytotoxicity against melanoma (27) and hepatocellular carcinoma cells (28). Concurrently, an alternate mechanism is proposed involving the endocytic pathway, wherein target cells internalize NKEVs carrying soluble FasL. Notably, the enrichment of FasL in NK-EVsIL-15 has been identified, and both BLI and MTT assays corroborate that NK-EVIL-15-mediated cell death is, in part, associated with the presence of FasL (29). However, a counter perspective contends that FasL might not substantially contribute to cytotoxicity, as some studies suggest its content in EV preparations ranks lowest among cytotoxic proteins. This viewpoint gains further support from protein correlations and Fas antibody blocking experiments, which collectively imply that FasL might not play a decisive role in the observed cytotoxic effects (16, 30).

#### 2.1.2 Cytokines

NKEV can inherit a series of cytokines produced by NK cells, such as interferon IFN-γ and TNF-α, and thus interact immunologically with other cells.

IFN-γ is a soluble dimeric cytokine and is a type II interferon with antiviral, antitumor, and immunomodulatory properties. Upon activation of IFN-γ receptor, a cascade is initiated wherein JAK1 and JAK2 undergo phosphorylation. The resultant phosphorylated STAT1 subsequently assembles into homodimers, translocating to the cell nucleus. In this nuclear milieu, these phosphorylated homodimers efficaciously exert their regulatory effects (31). Ample investigations have substantiated the presence of IFN-γ within EVs originating from NK cells. The consequential release of IFN-γ in close proximity to target cells by NKEVs has been demonstrated to curtail the proliferation and migration of endothelial cells, as supported by diverse studies (32, 33).

TNF-α is an inflammatory cytokine that plays a crucial role in immune regulation, cell proliferation, cell death, and morphogenesis through multiple signaling pathways (34). TNF-α has been found in EVs originating from diverse immune cells, including dendritic cells and macrophages (35, 36). Zhu et al. were the first to discover TNF-α in NK cell-derived EVs and confirmed the relevance of TNF-α to the cytotoxicity mediated by NKEVs in melanoma cells (27).

#### 2.1.3 Activating receptors

NKG2D, a type II transmembrane C-type lectin-like activating receptor, assumes significance as it forms homodimers and finds ubiquitous expression not only on NK cells but also on CD8^+^ T cells and a limited subset of CD4^+^ T cells (27). Research has shown that NKG2D is highly expressed on the membrane of NKEVs and is frequently used as a marker for EVs derived from NK cells (32). While the specific role of NKG2D on NKEVs is not yet clear, it has been confirmed that co-culturing cancer cells with NKEVs results in a significant decrease in apoptosis when anti-NKG2D antibodies is used (30).

DNAM-1, or CD226, represents a natural cytotoxicity receptor with broad expression encompassing T cells and a majority of quiescent NK cells. This receptor assumes a pivotal role in governing NK cell adhesion, cytotoxicity, and the facilitation of immune synapse formation (37, 38). Pace et al. blocked DNAM1 on the EV surface to inhibit the cytotoxic effects of NKEV; furthermore, DNAM1 may be ligand-bound to act through caspase-induced apoptosis (37, 39). However, in the apoptosis experiment using HCT116 spheroids, blocking DNAM-1 did not affect the apoptosis of recipient cells, possibly due to variations in ligand types and quantities on different target cells (30).

### 2.2 NKEVs regulate immune cells

In addition to cytotoxicity, NKEVs also exert immunomodulatory effects.The activation, inhibition of various immune cells and immune related modulators are directly or indirectly regulated by NKEV. Federici et al. observed that NKEVs exhibit the capacity to directly activate T cells. Moreover, these vesicles demonstrate the dual capability of inducing T cell proliferation, achieved either through direct stimulation or indirectly by elevating the expression of co-stimulatory molecules on monocytes (40). Jia et al. confirmed that NK-derived EVs can promote M1 polarization of macrophages, inhibit M2 polarization, thereby reducing bacterial load in mouse lung tissue to mitigate *Pseudomonas aeruginosa*-induced lung injury, and also decrease the percentage of neutrophils and lymphocytes in mouse lung tissue (41). Furthermore, the influence of NKEVs extends to both direct and indirect modulation of the function and activity of parent cells. Notably, an augmentation in the proportion of CD56^+^ NK cells is observed as an exemplar of the impact exerted by NKEVs (40). Not only does the number of NK cell subtypes change, but also their cytotoxicity and content are affected. NK cells subjected to treatment with NKEVs manifest not only a marked augmentation in cytotoxicity but also a concurrent elevation in the release of tumor necrosis factor (TNF) and perforin (42). Even more interestingly, various cytokine genes involved in regulating NK cell proliferation, cytotoxicity, and migration, such as the ligands for the chemokine receptor CXCR3, including CXCL9, CXCL10, and CXCL11, are significantly upregulated (42).

Furthermore, through some specific treatments of NKEVs, their immunomodulatory abilities can be enhanced. In a recent investigation, hydrophilic siRNA and hydrophobic photosensitizer Ce 6 were employed to modify NK-derived exosomes through light-activated silencing of NK (LASNEO). This innovative approach resulted in the induction of substantial photodynamic therapeutic effects, facilitated by the generation of reactive oxygen species (ROS) subsequent to laser irradiation. Notably, this intervention prompted the polarization of M1 tumor-associated macrophages and the maturation of dendritic cells within the tumor microenvironment (TME).Furthermore, the targeted application of siRNAs against PLK1 or PD-L1 elicited potent gene silencing in cancer cells. Intriguingly, the consequential downregulation of PD-L1 contributed to the restoration of immune surveillance by CD4^+^ T cells and CD8^+^ T cells within the TME. LASNEO displayed excellent anti-tumor effects by recruiting various types of immune cells (43).

### 2.3 EVs produced by memory-like NK cells

NK cells were previously believed to lack immune memory. However, increasing evidence suggests that NK cells can generate specific memory responses, acquiring “memory-like” functional characteristics, resulting in enhanced functional activity (44). Traditionally, the acquisition of memory-like traits by NK cells ensues in response to activation signals emanating from both target cells and the surrounding microenvironment. A case in point is the phenomenon observed in tissues, where resident decidual NK cells have the capacity to engender a distinctive and enriched NK cell subset during recurring pregnancies. This subset augmentation culminates in an enhanced production of IFN-γ and vascular endothelial growth factor (VEGF), thereby potentially contributing to improved placental development (45). Upon infection with mouse cytomegalovirus (MCMV),(NK cells in mice undergo a transition, acquiring adaptive immune features (46). Cultivating NK cells in the presence of artificial antigen-presenting cells (aAPC) and K562-mb IL-21 proves to be a transformative milieu, resulting in substantial expansion and activation of these cells. This orchestrated response is notably accompanied by a marked increase in the production of NK-EVs (47). Furthermore, EVs produced by memory-like NK cells exhibit greater toxicity towards cancer cells (48).

Furthermore, these memory-like NK cells can also produce EVs, enhancing their functional activity. EVs derived from NK cells previously exposed to neuroblastoma cells (NB), which express NCRs and activation signals, can activate resting human NK cells, enhancing their NK-mediated anti-NB tumor response (49). NK cells exposed in advance to EVs derived from CML cells exhibit higher gene expression levels of caspase 3 and P53 compared to the untreated EVs group, showing stronger cytotoxic effects on tumor cells (48).

Federici et al. proposed that there is no significant difference in the quantity and expression of surface markers on EVs produced by NK cells in the resting and activated states (50). However, current research indicates that memory-like NKEVs produced in response to cytokine stimulation appear to exhibit more effective cytotoxicity and anti-tumor effects.

Cytokine activation plays a crucial role in conferring memory-like characteristics to NK cells (51, 52). Stimulating NK cells with homeostatic and/or pro-inflammatory cytokines such as IL-2 and IL-15 enhances their effector functions, promotes anti-tumor immunity, and increases their persistence in the body (53).

In the study conducted by Zhu et al., it was discerned that the stimulation of an equivalent number of NK cells with IL-15 elicited a remarkable more than twofold augmentation in the overall production of extracellular vesicles (EVs). This increase extended not only to the quantity of EVs but also encompassed heightened protein content and particle numbers within these vesicles. Notably, IL-15-treated NKEVs (IL-15-treated NK-EVs) demonstrated a more potent tumor-targeting effect and an extended circulation period. These characteristics collectively resulted in a significant inhibition of the growth of heterotransplanted glioblastoma cells in murine models. Furthermore, the application of IL-15 was observed to correlate with an upregulation in the expression of Rab27a in NK cells. This observation suggests that IL-15 potentially exerts control over the cellular trafficking of Rab27a-specific cargoes, implicating a regulatory role in this particular pathway (29).

Enomoto et al. obtained similar conclusions when stimulating NK cells with IL-15, and IL-15 in combination with IL-21-induced EVs demonstrated stronger cytotoxic activity, even though the cytotoxicity of NK-92 cells was not enhanced under co-stimulation. This may be due to the NK-92 cells and the EVs they produce having different protein and RNA profiles, such as the enrichment of co-induced miR-146b and miR-23a, and the presence of CD226 (DNAM-1). Additionally, GZMB and GZMH were also co-induced by IL-15 and IL-21 (39).

In one study, it was revealed that the efficiency of EV production exhibited a notable increase when NK cells were subjected to co-stimulation with cytokines IL-15, IL-12, and IL-18, in comparison to stimulation with IL-15 alone. This heightened efficiency in co-stimulated EVs translated into a pronounced proclivity for spheroid apoptosis, particularly evident in the context of WM 9, OVCAR-3, and SK-RB-3 spheroids. Intriguingly, the cell lines WM9 and SK-RB-3, known for their resistance to NK cell-mediated killing, exhibited vulnerability to apoptosis induced by these co-stimulated EVs. This observation suggests a compelling prospect: that NK cell-derived extracellular vesicles possess the capability to target cells autonomously, irrespective of their donor cell origin (30).

Furthermore, EVs produced by stimulating NK cells with IL-1β did not show a significant change in total protein content. However, the expression of perforin significantly increased, and there was a dose-dependent enhancement of EVs in their inhibitory effect on endothelial cell proliferation and migration (33).

Moreover, it was observed that EVs generated through the stimulation of NK cells with IL-1β did not manifest a substantial alteration in their overall protein content (**Table 1**). However, the expression of perforin significantly increased, and there was a dose-dependent enhancement of EVs in their inhibitory effect on endothelial cell proliferation and migration.

**Table 1:** Extracellular vesicles produced by cytokine-activated memory-like NK cells.

Source of NK cells	Activation factor	Extraction method of Evs	Size Before using cytokines	Change in total number of Evs	Change in total amount of proteins	Functional changes	Effector molecules	Target cells	reference
NK-92MI	IL-15	Ultracentrifugation	106.9±21.6 nm(after use of IL-15118.2±20.3 nm)	More than twice as much as before	More than twice as much	Stronger anti-tumor activity *in vivo* and *ex vivo*, greater tumor targeting and longer circulation time *in vivo*	GZMB, perforin, and FasL were enriched; TRAIL, NKp30, and NKp44 increased	U87/MG, MDA-MB-231 and CAL-62	(29)
NK-92	IL-15	Ultracentrifugation	148.2 nm			Increase cytotoxicity	GZMB and GZMH slightly increased	K562, Jurkat, A549 and HeLa cells	(39)
	IL-21					No significant change in cytotoxicity			
	IL-15 and IL-21					Increase cytotoxicity	GZMB, GZMH, miR-146b and miR-23a enriched		
NK-92	IL-1β	Ultracentrifugation	210~490 nm		No increase	Inhibited endothelial cell migration, enhanced endothelial cell activation, and facilitated endothelial cell acquisition of pro-inflammatory and pro-coagulant phenotypes	Increased perforin and decreased granzyme B	EA.hy926 cell line endothelial cells	(33)
Primary NK cells	CD16	Test kit	60~125 nm(electron microscopy); 200 nm(particle size)			Weak cytotoxicity	Low levels of granzyme B and perforin	HCT116 colorectal cancer cells	(30)
	IL - 15			More than twice as much as CD16		Weaker than combined			
	IL-15, IL-12 and IL-18			More than IL-15 alone	2-fold higher than CD16-activated primary NK cells	Strongest cytotoxicity	Highest levels of granzyme B		
NK-92	IL-15			6-fold higher than CD16-activated primary NK cells	8-fold higher than CD16-activated primary NK cells	Stronger killing ability (weaker than primary NK)	High levels of granzyme A		
	IL-15, IL-12 and IL-18			7-fold higher than CD16-activated primary NK cells	4-fold higher than CD16-activated primary NK cells	Stronger killing ability (not as good asprimary NK)	Highest levels of granzyme A and high levels of granzyme B		
NKYG-1	IL-15			5-fold higher than CD16-activated primary NK cells	14-fold higher than CD16-activated primary NK cells	No cytotoxicity	Low levels of granzyme B and perforin		
	IL-15, IL-12 and IL-18			5-fold higher than CD16-activated primary NK cells	7-fold higher than CD16-activated primary NK cells				

## 3 NK cell source

**Figure 2:**
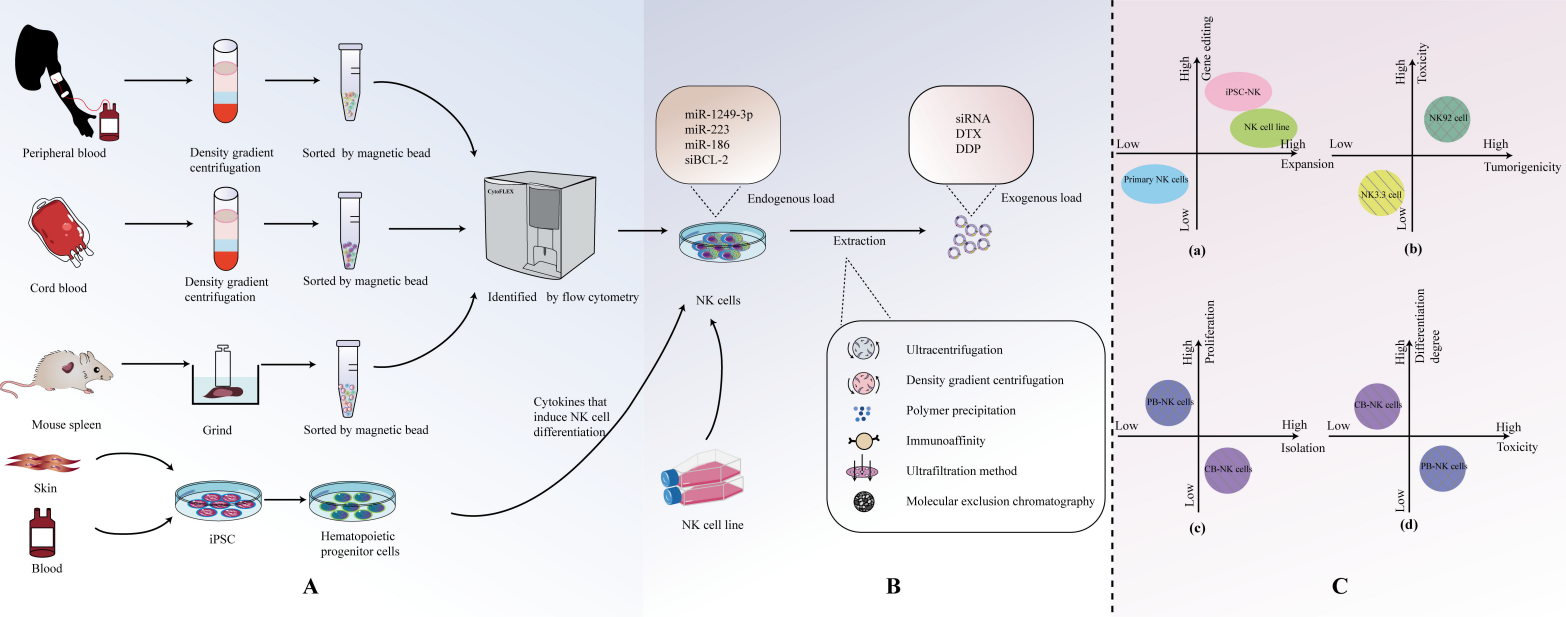
**(A)** Origin and isolation of NK cells. **(B)** Isolation of NKEVs. **(C)** Comparison of NK cell-derived EVS from different sources.

There are various options for the sources of NK cells used to generate EVs (**Figure 2A**). Currently, common sources for primary NK cells include peripheral blood-derived NK cells (PB-NK), cord blood-derived NK cells (CB-NK), and splenic NK cells from mice. In addition, immortalized NK cell lines, such as the NK-92 cell line and the NK3.3 cell line, are also commonly used. Recently, NK cells derived from induced pluripotent stem cells (iPSC) and highly efficient chimeric antigen receptor (CAR)-armed NK cells have demonstrated effective expansion capabilities, making them potential sources of EVs. NK cell-derived EVs from different sources exhibit distinct characteristics in terms of isolation and extraction, cytotoxicity, and safety (**Figure 2C**).

### 3.1 Primary NK cells

The number of NKEVs (NK cell-derived extracellular vesicles) in human blood is low, and there are many other cell-derived EVs, making isolation challenging (37, 50). Therefore, a preferable approach, compared to directly extracting NKEVs from blood, is to first obtain primary NK cells and then extract NKEVs. Human peripheral blood emerges as the most readily accessible source for procuring primary NK cells. Typically, single-nucleated cells are separated from the blood using density gradient centrifugation. NK cell populations are then obtained through flow cytometry (54). However, NK cells in peripheral blood are mature, and their viability decreases when frozen, making long-term storage difficult. Umbilical cord blood contains an abundant population of immature NK cells that are highly tolerant to freezing and exhibit good homing to the bone marrow (55). Furthermore, NK cells expanded from cord blood have higher proliferation capacity and lower cytotoxicity compared to NK cells obtained from peripheral blood (56–58). In a study by Luo et al., the successful isolation of cord blood mononuclear cells (CBMC) from cord blood was achieved through the implementation of density gradient centrifugation. Subsequently, NK cells were co-stimulated using IL-2 and irradiated K562-engineered cells. This tailored approach resulted in a noteworthy expansion of NK cells and facilitated the subsequent isolation of a substantial quantity of highly cytotoxic and high-quality NK-EVs (42). For commonly used laboratory animals like C57BL6 mice, obtaining blood samples is limited, and spleen tissue is often used as the source for NK cells. Spleen tissue is processed into a single-cell suspension through grinding, and then CD3- CD49b+ cell populations are obtained using flow cytometry to isolate NK cells (59).

### 3.2 NK cell line

NK-92 is the most commonly used NK cell line. In comparison to the complex process of collecting NK cells from peripheral blood mononuclear cells (PBMCs) and activating them for seven days, NK-92EV (extracellular vesicles secreted by the NK-92 cell line) can be rapidly isolated and utilized for clinical immunotherapy (27, 50, 60). It is currently the only NK cell line approved by the FDA for clinical applications and is the most widely used NK cell line in experimental research. NK-92 is a NK tumor cell line derived from a non-Hodgkin’s lymphoma patient (61), and EVs from these transformed/tumor cells may carry cargo specific to cells capable of altering receptor cells or causing adverse effects (62, 63). However, studies have indicated that the protein levels of cytotoxic proteins in EVs derived from PB-NK cells are generally higher than those from NK-92 sources (16).

NK3.3, a non-tumorogenic NK cell line originating from the peripheral blood of a healthy donor, presents a distinct advantage. This unique lineage alleviates the requirement for identifying and securing consent from numerous healthy peripheral blood donors, concurrently mitigating concerns related to donor variability. Importantly, the utilization of NK3.3 circumvents the potential introduction of oncogenic elements that may be associated with the use of transformed NK-92 cells. Furthermore, this cell line demonstrates the capacity for extensive expansion in substantial quantities, contributing to its utility in research and applications requiring large-scale production (64). Although research has shown that NK-92 cells are more effective than NK3.3 cells in lysing the K562 leukemia cell line, in Cochran et al.’s study, EVs sourced from NK3.3 cells have demonstrated the capacity to instigate morphological transformations and modulate protein expression patterns pertinent to apoptosis induction in diverse cancer cells. This consequential effect manifests as a potent inhibition of tumor proliferation, accompanied by robust cytotoxicity specifically targeted at K562 cells. Importantly, these discernible impacts on cancer cells do not extend to exert any influence on the growth or viability of non-tumorogenic normal cells (64, 65). Hence, NK3.3 emerges as a promising candidate with the potential to function as an efficacious, safe, and universally applicable immunotherapeutic agent.

### 3.3 iPSC and car-NK cells

NK cells derived from induced pluripotent stem cells (iPSC) have been shown to be superior in cellular therapy compared to primary NK cells and the NK-92 cell line. The therapeutic properties of iPSC-derived cardiomyocyte (66)-EVs, MSC (67)-EVs, and iPSC (68)-EVs have been confirmed. However, further research on their extracellular vesicles (EVs) has not been conducted yet, and this may be a highly promising research direction that deserves further exploration (69, 70). Moreover, the recent advancement in the creation of CAR-equipped NK cells, strategically designed to target specific tumor antigens, marks a sophisticated and potent avenue for EV sourcing. These EVs have the potential for higher specificity in targeting tumor cells (71).

## 4 Extraction of NKEVs

Currently, the methods for EVs are continually evolving and being updated. Commonly used methods include ultracentrifugation, density gradient centrifugation, polymer precipitation, immunoaffinity methods, ultrafiltration, and size exclusion chromatography (**Figure 2B**). In fact, the purity of isolated EVs often comes at the cost of sacrificing factors such as cost, yield, scalability, and therapeutic efficacy. If one aims to enrich more EVs in a specific isolation, it inevitably requires more time, labor, and cost (55, 72).

In recent years, some new extraction methods have gradually become research hotspots, such as microfluidic devices, which can efficiently and precisely separate particles of micrometer or nanometer size in a given volume of fluid (73). Due to their miniaturization, integration, high-throughput capacity, and low time consumption, microfluidic devices hold great promise for improving recovery rates, reducing sample volumes, and shortening processing times. In consideration of the synergistic benefits arising from the integration of microfluidic devices and chemical release strategies, Kang et al. introduced a refined microfluidic platform incorporating anti- NK cell antibody functionality, denoted as the NK-go chip. This innovative platform leverages biocompatible graphene oxide for the capture of NK cell-derived exosomes (NK-Exos) during short-term culture. Additionally, it employs anti-CD63 magnetic beads (ExoBeads) to facilitate the subsequent recovery and purification processes. The study found that the highest exosome purity is obtained with a 12-hour incubation on the chip (74).

Wu et al. have developed a seesaw-motion bioreactor (SMB) system with continuous fluid flow, which not only expands the production of extracellular vesicles (EVs) by increasing the yield of EVs per cell but also achieves scalable EV production by increasing the working volume and cell density of the cell culture medium during continuous-flow cell culture. Through *in vivo* and *in vitro* experiments, it has been demonstrated that the toxicity of NKEVs produced in this manner is not significantly altered compared to static conditions (75).

## 5 Therapeutic potential of NKEVs-based delivery platforms for the treatment of chronic diseases

Over the past few years, exosomal therapies have made remarkable progress, and the ability to leverage cell-to-cell transfer of information is increasingly becoming a focus of chronic disease research. More importantly, EVs are loaded with a wide range of bioactive molecules from the parent cell, mainly drugs, lipids, proteins and nucleic acids (DNA, coding and non-coding RNA) (76) (**Table 2**). These loads can be introduced before or after exosome isolation. Pre-isolation loading methods Refers to the introduction of therapeutic molecules into parental cells prior to EV production so that they are encapsulated prior to EV biogenesis (82) (**Table 3**). Currently, NK cell-derived EVs have received extensive attention as delivery vectors for miRNAs or drugs, and there is a large scope for development in the transport of siRNAs. NKEVs emerge as inherently advantageous drug carriers, distinguished by their elevated biocompatibility, diminished immunogenicity, and the capacity to traverse the blood-brain barrier. Noteworthy in their role as cargo transporters, NKEVs exhibit intrinsic targeting capabilities and cytotoxicity during transit, thereby eliciting potent killing effects. Moreover, the regulatory influence of NKEVs extends to the modulation of signaling pathways within recipient cells, thereby orchestrating anti-tumor functions through the efficient delivery of cargo. This multifaceted potential positions NKEVs as a promising avenue for therapeutic interventions in chronic diseases, encompassing conditions such as cancer, diabetes, depression, and immune disorders (84).

**Table 2:** Cargo loaded by NKEVs.

Source of NK cells	Extraction method of Evs	Engineering approach	Target cells	Function	reference
NK-92	Differential ultracentrifugation	Electroporation loading paclitaxel PTX	Human breast cancer MCF-7 cells	Increased cellular uptake of drugs, promoting apoptosis	(77)
PB-NK, CB-NK	Differential ultracentrifugation	Electroporation loading of cisplatin	Human ovarian cancer cell lines SKOV3, OV-90, COC1/DDP	Promoted apoptosis and improved drug resistance	(42)
NK-92MI	Differential ultracentrifugation	Electroporation loading of siRNA targeting PLK1 (siPLK1), modified with photosensitizer Ce6	Hepatocellular Carcinoma HepG2-Luc cell line, Colorectal carcinoma CT26 cell line, Murine macrophage RAW264.7 cell line	Promoted polarization of M1 tumor-associated macrophages and DC cell maturation	(43)
NK-92MI	Test kit	siBCL-2 transfection into NK cells by virus	ER+ breast cancer cells HEK293T cells	Activated mitochondria-dependent apoptosis	(78)
NK92-MI	Differential ultracentrifugation	miR-223 transfected into NK cells	Human hepatic stellate cells-LX-2	Targeted ATG7, inhibited TGF-β1-induced autophagy and attenuated TGF-β1-induced stellate cell activation	(79)
PB-NK	Differential ultracentrifugation		Human PC cells (MiaPaCa-2 and PANC-1)	Inhibited cell proliferation, migration and invasion	(80)
PB-NK	High Performance Liquid Chromatography	miR-186 transfected into NK cells	Neuroblastoma cell line	Inhibited tumorigenic potential of adult neuroblastoma and prevented TGFβ1-dependent inhibition of NK cells	(81)
PB-NK	Differential ultracentrifugation	Polyamidoamine hybridization of NKEVs with reproducible let-7a (membrane fusion)	Human adult neuroblastoma CHLA-255 cells (MDA-MB-231-luc, CHLA-255-luc cells)	Inhibited tumor growth	(54)

**Table 3:** Summary of studies on the NKEV drug delivery platform.

Source of NK cells	Extraction method of Evs	Size	Markers of Evs	Use Pathway of Evs	Loading Substances	Loading Method	Target Cells	Mechanism and Function	reference
NK-92	Differential ultracentrifugation	80~110nm	CD63, protein Alix, TSG101	Intravenous injection	Paclitaxel (PTX)	Electroporation	Human breast cancer MCF-7 cells	Inhibited cell proliferation and induced apoptosis more significantly than free PTX	(77)
NK-92MI	Differential ultracentrifugation	0~150nm	ALIX, CD63		MiR-223	Cell transfection	Human hepatic stellate cells-LX-2	Targeted ATG7, inhibited TGF-β1-induced autophagy and attenuated stellate cell activation	(79)
NK-92MI	CD63 Triisosexual ™ exosome detection kit	115.8~128.9 nm	CD63		SiBCL-2	Cell transfection	Breast cancer cells(MEC-1, MCF-7, T-47D, SKBR3, MDA-MB-MB-231)	Promoted apoptosis	(78)
PB-NK	Differential ultracentrifugation		D63, TSG101	Intravenous injection	MiR-3607-3p	Liposome transfection	Human PC cells (MiaPaCa-2, PANC-1)	Targeted IL-26, inhibited cell proliferation, migration and invasion.	(80)
Mouse spleen CD3-CD49b+ cells	Test kit	50-150 nm	CD81, CD63	Intravenous injection	Cy3-miR-207	Cell transfection	Astrocytes	Targeted Tril to exert antidepressant effects	(59)
PB-NK	Size exclusion chromatography(SEC)	92.45 nm	CD56(CD81, Calnexin, TSG101)		MicroRNA (miR)-186		Neuroblastoma cell lines	Inhibited tumorigenic potential of neuronal neoplasms, prevented TGFβ1-dependent inhibition of NK cells	(81)
Mouse splenic lymphocytes	Ultracentrifugation	30~150 nm	HSP70, CD63, TSG101, CD9	Tail vein injection	MiR-1249-3p	Cell transfection	Adipocytes and hepatocytes (3T3-L1 adipocytes, AML12 adipocytes)	Targeted SKOR1, inhibit TLR4/NF-κB pathway, enhanced insulin sensitivity, reduced inflammation in adipocytes and hepatocytes	(83)
PB-NK, CB-NK	Differential ultracentrifugation	73.2 ± 28.5 nm	CD81, CD63, TSG101		Cisplatin	Electroporation	Human ovarian cancer cell lines (SKOV3, OV-90, COC1/DDP)	Killed OC cells and sensitized tumor cells to DDP	(42)
NK-92MI	Differential ultracentrifugation	120 nm	CD9, CD63, CD81, TSG101	Intratumoral injection	SiRNA targeting PLK1 (siPLK1)	Electroporation	Hepatocellular Carcinoma HepG2-Luc cell line, Colorectal carcinoma CT26 cell line, Murine macrophage RAW264.7 cell line	Promoted polarization of M1 tumor-associated macrophages and DC cell maturation	(43)

### 5.1 NKEVs and breast cancer

The inclusions loading capacity and nano-size of EVs, coupled with the membrane proteins inherited from NK cells, so NKEV provides an important solution as a delivery strategy to efficiently deliver small molecule nucleic acids to breast cancer cells. As an illustrative instance, the NK cell line NK92MI underwent lentiviral transduction for the purpose of expressing small interfering RNAs targeting BCL-2 (siBCL-2) within EVs denoted as NKExos. This strategic modification ensured the encapsulation of siBCL-2 within NKExos without imposing a substantial impact on NK cell viability or effector function (78). Subsequently, siBCL-2NKExos targeting BCL-2 enhanced intrinsic apoptosis in breast cancer cells without affecting non-malignant cells. Meanwhile, NKEV can also deliver other non-nucleic acid cargoes. Han et al. extracted EVs from NK-92 cells and prepared paclitaxel PTX-NK-Exos delivery system by electroporation, which targeted human breast cancer MCF-7 cells, effectively inhibited the proliferation of tumor cells and induced apoptosis, thus exerting anti-tumor effects (77). Not only that, NKEV delivery of iron death inducer induced RSL3 leading to intracellular lipid peroxidation in breast cancer cells, resulting in iron death (85).

### 5.2 NKEVs and other cancers

A plethora of studies has substantiated the involvement of EVs in cancer development, underscoring their pivotal role in the regulation of tumor growth, invasion, and metastasis. These effects are attributed to the dynamic influence of EVs on the tumor microenvironment and their adept modulation of the immune response (86, 87). EVs derived from NK cells have a certain targeting ability. NKEVs can distinguish between transformed and non-transformed cells, and NK-EVs can effectively kill tumor cells. This highlights a potential and interesting application of NKEVs in cancer treatment (37). Kim et al. found that NKEVs have a strong targeting effect and significant toxicity on solid tumors in a primary liver cancer mouse model but do not affect the activity and apoptosis of normal liver cells (28). Currently, gliomas (LGG) have a poor prognosis with a lack of specific biomarkers. While there exists ongoing research aimed at constructing prognostic markers for lower-grade gliomas (LGG) linked to potential factors such as immunogenic cell death (88), inflammatory signaling (89), or long non-coding RNAs (90), the pervasive and inherent capability of exosomes to traverse the blood-brain barrier (BBB) presents an avenue of greater promise in the realms of diagnosis, targeted treatment, and prognosis assessment for brain diseases. As highlighted by Zhu et al., NK-EVs containing IL-15 exhibit heightened cytolytic activity against various human cancer cell lines, including glioblastoma, breast cancer, and thyroid cancer. This enhanced cytotoxicity is accompanied by an upregulation of molecules associated with NK cell cytotoxicity. Importantly, NK-EVs do not exhibit significant toxicity against normal cells or murine models, emphasizing their potential as a safe and effective therapeutic modality (29).

However, the targeting mechanisms of NKEVs are not yet clear, but some believe that surface receptors on the vesicles such as TRAIL, NKp30, and NKp44, or adhesion molecules such as lymphocyte function-associated antigen (LFA)-1/intercellular adhesion molecule (ICAM)-1, are involved in the recognition and targeting of tumor cells (29, 91).

NKEV delivers miRNAs for the treatment of a variety of cancers, mainly including its mechanism of action includes direct binding to proteins, preventing their binding to receptors as ligands, and triggering downstream signaling, thus altering the activity of the target cells. Neviani et al. primarily utilized a liposomal preparation, anionic liposome nanoparticles (lypopolyplex nanoparticles, the NP) loaded with miR-186 mimics or controls, exposed to PBMC-isolated NK cells, and EVs isolated from NK cell supernatants delivered mature miRNAs targeting and impairing neuroblastoma cell survival and migration, while resisting NK cell inhibition, to achieve inhibition of the tumorigenic potential of the cells (81). Cytokines can also serve as miRNA targets. SUN et al. found that NK cells isolated from PBMC, which derived EVs, directly targeted IL-26 via miR-3607-3p, in which LNM^+^PC patients (pancreatic cancer patients with lymph node metastasis) had higher levels of IL-26 than the control group, and that IL-26 may play a role in the reduction of metastasis of tumor cells, thereby inhibiting their malignant transformation (80). Contemplating the gene-silencing efficacy inherent to siRNA (92), the intercellular transferability of EVs between tumor cells and immune cells (93), and the tumor-homing proficiency exhibited by NKEVs (94), it is plausible to envisage that NKEV possesses the potential to orchestrate the modulation of immune cell activity. This may be achieved through the mediation of siRNA, consequently impeding the initiation and advancement of tumors. NKEVs modified with the hydrophobic photosensitizer Ce 6, loaded with hydrophilic siRNAs through electroporation, and subsequently subjected to laser activation, instigated substantial photodynamic therapeutic effects. This intervention not only facilitated the polarization of M1 tumor-associated macrophages and the maturation of dendritic cells (DC) within the tumor microenvironment (TME) but also orchestrated the recruitment of various immune cell types. Impressively, these tailored NKEVs demonstrated outstanding efficacy in exerting anti-hepatic tumor cell effects (43).

Most delivery platforms for cancer treatment based on Nanoscale Extracellular Vesicles (NKEV) are centered around nucleic acid therapies, particularly miRNA. However, theoretically, there may be a broader range of applications. As one of the three major gynecological cancers, the treatment of ovarian cancer using radiotherapy and chemotherapy is hampered by issues such as drug resistance and long-term complications. Considering the toxicity and loading capacity of Nanoscale Extracellular Vesicles (NKEVs), along with the effectiveness of immunotherapy, combining NKEVs with immunotherapy for anticancer treatment may have potential applications (95). Cisplatin can inhibit tumor proliferation. Luo et al. loaded cisplatin into eNK-EXO, enhancing drug sensitivity in cisplatin-resistant ovarian cancer cells. Additionally, it can activate NK cells in the immunosuppressive tumor microenvironment, ultimately achieving an anti-ovarian cancer cell effect (42). In conclusion, utilizing extracellular vesicles derived from natural killer (NK) cells to load chemotherapeutic agents can enhance the uptake by solid tumors, thereby achieving a more precise and effective drug delivery.

### 5.3 NKEVs and other chronic diseases

Due to the cell-permeable capacity of exosomes and their ability to cross the blood-brain barrier, NKEV may be a promising strategy for the treatment of psychiatric or metabolic chronic diseases. As an exemplar, EVs derived from NK cells, isolated from mouse spleens, exhibited the capacity to traverse the blood-brain barrier. These EVs were subsequently internalized by astrocytes, conveying miR-207, which directly targeted proteins interacting with TLR4-Tril complexes, consequently inhibiting the NF-κB signaling pathway. This orchestrated molecular modulation led to a diminished release of pro-inflammatory cytokines. Notably, such intervention translated into a reduction in stress-related symptoms, including locomotor retardation, thereby eliciting antidepressant effects in the mice (59). 2021 wang et al. After co-culturing mouse splenic lymphocyte NK cells transfected with miR-1249-3p with 3T3 - L1 adipocytes or AML12 cells, miR-1249-3p from EV of the former origin inhibited the NF-κB signaling pathway, with novel roles in insulin resistance mitigation and attenuation of inflammatory response, a common dysfunction in patients with type 2 diabetes (83). However, the role of NKEV in immune chronic diseases is easily overlooked, and it has been demonstrated that NKEV exhibits a dose-dependent killing effect for K562 cell lines or Jurkat cell lines derived from chronic granulocytic leukemia or acute T-cell leukemia (39).

## 6 NKEVs in clinical diagnosis and treatment

In addition to being widely recognized as a nano-scale carrier transport platform, they also have a wide range of applications in clinical diagnosis and treatment. From the perspective of NK cells, on one hand, NK cells, as important effector cells of the innate immune system, can rapidly identify and eliminate heterogeneous cells such as virus-infected cells, tumor cells, and respond to the early pathological conditions of the body. On the other hand, NK cells differentiated in different tissue types exhibit a high degree of heterogeneity, and even within the same organ or tissue, NK cells can have different phenotypic characteristics and functions in different functional states. The high complexity of cell differentiation within tissues makes NK cells a potential specific indicator of the body’s pathological functional state, especially in the early stages of disease. At present, clinical data has shown that the abundance of NK cells in the TME predicts a better prognosis for patients with various cancers, such as hepatocellular carcinoma (HCC), melanoma, and others. Simultaneously, researchers have established a signature of NK cell (NRG)-related genes to assess the immunotherapeutic efficacy in head and neck squamous cell carcinoma (96). Because EVs can inherit various characteristics and specific biomolecules from parent cells, it is one of the reasons for the heterogeneity of EVs. Therefore, NKEVs inherit the potential of NK cells as specific biomarkers. As early as 2012, it was discovered that in pre-eclampsia, the number of microvesicles in peripheral blood would undergo changes, and among them, there were fewer microvesicles formed by NK cells (97). The correlation between the content of EVs released by NK cells in the blood and the development of the disease reveals the potential clinical application value of NKEVs in disease diagnosis and treatment.

In recent years, through the application of microfluidic systems for NKEV extraction, it has been discovered that changes in NKEV concentration are positively correlated with the number of circulating tumor cells (CTC) in non-small cell lung cancer (NSCLC) patients (74). CTCs, representing subclones with a high metastatic propensity, are used in liquid biopsies for cancer. They provide more information than traditional tissue biopsies through phenotype and molecular analysis and can serve as biological markers of interest in precision cancer treatment. In 2017, the National Comprehensive Cancer Network (NCCN) in the United States included circulating tumor cells (CTC) in the clinical guidelines for breast cancer malignant tumor staging (TNM staging). Given the diagnostic significance of CTC in cancer and the robust association between NKEV and CTC counts, a compelling prospect emerges—namely, that the identification of NKEVs could offer a personalized approach to disease diagnosis and treatment. This notion supplements and amplifies the predictive capacity of CTCs in delineating patient prognosis. In a noteworthy development, Deeptha et al. engineered a highly sensitive, highly specific, and straightforward GBM ImmunoProfiler platform. This platform harnessed an expandable ultrafast laser multiphoton ionization mechanism to scrutinize serum samples from glioblastoma (GBM) patients, enabling the tracking of NKEV expression in the circulation alongside immune checkpoint markers, namely PDL-1 and cytotoxic T-lymphocyte-associated protein 4 (CTLA4). The clinical validation of this cutting-edge technology furnishes robust evidence advocating for the utilization of NKEVs as a diagnostic and therapeutic tool in minimally invasive biopsies (98).

## 7 Discussion and future

NK cells are important immune cells in the body, and their EVs have similar characteristics to the parental cells. Compared with other immune cells, such as T cells, T cells need expensive and time-consuming engineering and expansion of T cell processes, as well as the therapeutic effect limitation caused by low expression of major histocompatibility complex (MHC) on tumor cells. NK cell therapy is independent of antigen presentation and can be better controlled to reduce the risk of cytokine storm.

EV has many advantages, such as biocompatibility, blood-brain barrier penetration, small size, and suitable for infiltrating solid tumors (59, 81). The cytotoxicity of NK cells to a variety of tumors is inhibited by acidic extracellular pH, which inhibits the release of perforin/granzyme containing particles and fas/fasl interaction (50, 99). The promotion of EV accumulation and delivery is notably facilitated by acidity. Within the acidic tumor microenvironment, this distinctive milieu actively encourages the uptake of EVs by tumor cells. This phenomenon is driven by the compelling influence of low pH, which not only attracts EVs but also fosters the facilitation of membrane fusion processes (27, 100). Moreover, NK extracellular vesicles (NK exo) have natural tumor targeting ability and immune regulation ability, and are ideal molecular carriers, which can effectively transmit drugs or signal molecules to tumor cells or immune cells, thereby enhancing the anti-tumor effect. In addition, the exosome production of NK92 cells under hypoxia treatment increased (101); The hypoxic milieu proves conducive to both the accumulation and delivery of exosomes. This advantageous effect arises from the low oxygen concentration within the environment, a factor that attracts exosomes and facilitates their membrane fusion processes (102).

Even though NKEVs (NK cell-derived Extracellular Vesicles) have been shown to have some degree of specificity towards tumor sites, the mechanism of EVs targeting specific cells and the precise delivery of therapeutic EVs to target cells remain a yet-to-be-solved and critically important issue. Addressing this issue could enable us to utilize EVs as a means of delivering more therapeutic EVs to target cells and avoiding unwanted side effects. In recent years, engineered EVs have gradually come into focus, and surface modifications of EVs, including chemical modifications and genetic engineering, have significantly enhanced their tumor-targeting capabilities. The development of EV-nanomedicine co-delivery systems, such as liposomes, can further enhance the loading efficiency of EVs while ensuring their targeting specificity. This approach has also been validated in EVs derived from NK cells (54, 103). Microfluidics and lab-on-a-chip technologies make it possible to control the size of liposomes, and freeze-thaw cycles appear to be advantageous for the fusion of EVs with liposomes. These advancements make EV-nanomedicine delivery systems even more promising in terms of their potential for targeted drug delivery (104, 105).

## Author contributions

HC: Conceptualization, Writing – review & editing. SH: Conceptualization, Data curation, Writing – original draft. LS: Data curation, Visualization, Writing – original draft. HH: Writing – original draft. HL: Writing – original draft. JX: Writing – original draft. XG: Writing – original draft. QW: Conceptualization, Writing – review & editing. GY: Conceptualization, Writing – review & editing.
